# Simultaneous Inhibition of PI3K and PAK in Preclinical Models of Neurofibromatosis Type 2-related Schwannomatosis

**DOI:** 10.21203/rs.3.rs-3405297/v1

**Published:** 2023-10-16

**Authors:** Cristina Fernandez-Valle, Anna Nagel, Julianne Huegel, Alejandra Petrilli, Rosa Rosario, Berta Victoria, Haley Hardin

**Affiliations:** University of Central Florida

**Keywords:** Phosphoinositol-3-kinase, p21-activated kinase, schwannoma, nerve allograft, merlin, preclinical model, schwannomatosis

## Abstract

Neurofibromatosis Type 2 (NF2)-related schwannomatosis is a genetic disorder that causes development of multiple types of nervous system tumors. The primary and diagnostic tumor type is bilateral vestibular schwannoma. There is no cure or drug therapy for NF2. Recommended treatments include surgical resection and radiation, both of which can leave patients with severe neurological deficits or increase the risk of future malignant tumors. Results of our previous pilot high-throughput drug screen identified phosphoinositide 3-kinase (PI3K) inhibitors as strong candidates based on loss of viability of mouse merlin-deficient Schwann cells (MD-SCs). Here we used novel human schwannoma model cells to conduct combination drug screens. We identified a class I PI3K inhibitor, pictilisib and p21 activated kinase (PAK) inhibitor, PF-3758309 as the top combination due to high synergy in cell viability assays. Both single and combination therapies significantly reduced growth of mouse MD-SCs in an orthotopic allograft mouse model. The inhibitor combination promoted cell cycle arrest and apoptosis in mouse merlin-deficient Schwann (MD-SCs) cells and cell cycle arrest in human MD-SCs. This study identifies the PI3K and PAK pathways as potential targets for combination drug treatment of NF2-related schwannomatosis.

## Introduction

Neurofibromatosis Type 2 (NF2)-related schwannomatosis is a rare autosomal dominant disorder that causes formation of multiple tumor types in the nervous system(1). Although the primary and diagnostic tumor type is bilateral vestibular schwannoma (VS), patients simultaneously develop other schwannomas, meningiomas, and ependymomas(2). Pathogenic variants in *NF2* inactivate the merlin tumor suppressor, a scaffold protein controlling downstream signaling from numerous cell surface receptors that stimulate Schwann cell proliferation and/or survival(3, 4). Individuals with NF2 undergo regular monitoring and tumors are treated, when necessary, with surgery, radiation, or experimental drug therapies(5–7). There is a great need for an effective and well-tolerated drug therapy for this patient population.

NF2 pathogenic variants have been identified in spontaneously occurring schwannomas and meningiomas, as well as many types of cancer including mesothelioma, glioma multiforme, breast, colorectal, skin, hepatic and prostate(8–10). Population studies suggest that tumors with sporadic *NF2* mutations could occur in up to 1 in 300 people(11). Although the role of merlin inactivation has not been well characterized in these other cancers, *NF2* status may be relevant for prognosis and future chemotherapeutic approaches.

Among the many merlin-dependent signaling pathways that control Schwann cell proliferation and survival is the PI3K pathway where merlin was demonstrated to bind the PI3K enhancer PIKE-L to inhibit PI3K signaling(8). Our previous pilot high-throughput screening study identified PI3K inhibitors as drug candidates based on their efficacy to reduce viability of mouse merlin-deficient Schwann cells(12). Preliminary screening of 193 compounds alone and in combination has been conducted on our mouse and human merlin-deficient Schwann cells by the SYNODOS Consortium. Multiple PI3K inhibitors met criteria for efficacy and/or synergy(13, 14). Although targeted single-agent chemotherapeutics can inhibit tumor growth, drug resistance is common due to activation of alternative signaling pathways that sustain cell survival and proliferation(15)^,^(16). The purpose of this study was to identify drug combinations that co-targeted the PI3K pathway and secondary pathways using cell-based high-throughput screens and to confirm the efficacy of the most successful combination in an NF2-specific animal model.

Utilizing a synergistic drug screening approach, we identified a novel drug combination targeting PI3K and P21-associated kinase (PAK), confirmed its efficacy in an animal model of NF2-related schwannomatosis, and explored the mechanism through which these inhibitors decreased mouse and human MD-SCs proliferation and survival. Moreover, by identifying known merlin interactors as drug targets for schwannomas, we confirm the utility of our screening strategy and human schwannoma model cell lines for future identification of novel targets.

## Materials & Methods

### Cell Culture

Primary human fetal Schwann cells (SC), HS11 and HS13 were acquired from SciencCell Research Laboratories (Carlsbad, CA, USA). *NF2*-shRNA treatment was utilized to suppress merlin expression in HS11 cells to create two merlin-deficient SC (MD-SC) lines, HS01 and HS02. The HS03 and HS05 MD-SC lines were created by CRISPR/Cas9 with NF2sg1 (sequence: AAACATCTCGTACAGTGACA, provided by Broad Institute) in HS13 cells. Development and characterization of these cell lines, including loss of merlin, was previously described(8). Human SCs were cultured on CellBIND Corning dishes (Corning, Corning, NY, USA) in complete Schwann cell media (SCM, basal Schwann cell medium plus 5% fetal bovine serum, Schwann cell growth supplements, 100 Units/mL penicillin, and 100 μg/mL streptomycin). Mouse MD-SCs (MTC-10) were generated and characterized in 2010 and then underwent lentiviral luciferase transduction(8, 17). Mouse SCs were cultured on CellBIND Corning dishes in N2 medium (F12, Dulbecco’s modified Eagle medium, and 1% N2 supplement, Gibco, ThermoFisher Scientific, Waltham, MA USA). Luciferase expression was confirmed prior to in vivo use by Western blot. All cells were routinely tested for *mycoplasma* contamination (LookOut Mycoplasma PCR Detection Kit; Sigma Aldrich, St Luis, MO, USA).

### Cell Viability and Synergy Analysis

Drugs used in this study are listed with their targets in Table S1. All drugs were obtained from SelleckChem (Houston, TX, USA), apart from (−)-B-Hydrastine (Santa Cruz Biotechnology, Dallas, TX, USA). Drugs were dissolved in DMSO (10 mM stock). MD-SCs were seeded in 384-well CellBind Corning plates at 1000 cells/well in phenol red-free media. After attachment, cells were treated with drugs at 10 concentrations in a 10×10 matrix. Mouse cells were treated for 48h and human cells for 72h prior to assessment of cell viability with CellTiter-Fluor Assay (Promega, Madison, WI, USA). All drugs were tested in at least two human MD-SC lines. Follow up testing was performed on a subset of combinations using the mouse MD-SC line. Matrix combination data was uploaded into the Combenefit software system to assess drug synergy using three methods (Loewe, Bliss, and HSA)(18).

### Cell Cycle Analysis

HS01 cells were seeded at 125,000 cells/well in a 6-well CellBIND Corning plate and al-lowed to attach overnight. Cells were then treated with 0.001% DMSO, 2.5 μM pictilisib, 150 nM PF-3748309, or a combination of the two drugs for 24h. EdU (10 μM) (Click-iT EdU kit; Molecular Probes, ThermoFisher) was added during the last 3h. Cells were collected with 0.05% trypsin, stained with violet live/dead stain (ThermoFisher Scientific), and permeabilized. DNA labeling with FxCycle stain (ThermoFisher Scientific) was performed according to manufacturer’s instructions. Cell populations were identified by flow cytometry on CytoFlex (Beckman Coulter, Brea, CA, USA) and analyzed by CytExpert software (Beckman Coulter).

### Cell Death Assay (Incucyte)

Human HS11, HS01, HS02, HS13 and HS05 and mouse MTC-10 cells (1,000 cells/well) were seeded in growth medium in three replicate wells/condition of a 384-well CellBIND Corning plate. After ~ 6h, drugs (diluted in 0.001% DMSO) at three increasing concentrations (EC50 as well as lower and higher concentrations) and Incucyte^®^ Caspase-3/7 Green Apoptosis Assay Reagent (Sartorius, Gottingen, Germany) were added to the wells. Wells were imaged using the Incucyte^®^ S5 Live-Cell Analysis system (Sartorius) for 24–72h. Phase and green fluorescent images were collected every 2h and analyzed using the integrated basic analyzer software.

### Apoptosis Array Assay

Human HS02 MD-SCs and mouse MTC-10 (MS01) MD-SCs cell lines were used. Briefly, cells were seeded on 10cm plates and treated with DMSO (0.001%) or pictilisib (2.5 μM) for 6h. This time was selected based on previous Incucyte data and coincided with MTC-10 cells started to round up and detach. Cells were lysed and procedure was performed according to the Proteome Profiler Human or Mouse Apoptosis Array Kit (Cat No: ARY009 for Human, Cat No: ARY031 for Mouse, Biotechne, Minneapolis, MA, USA). Arrays were visualized using the Jess System (Biotechne), densitometry was measured and normalized to reference spots using ImageJ Software (NIH, Bethesda, MD, USA). Calculations were carried out using Prism 9 Software (GraphPad, Boston, MA, USA).

### Pharmacokinetic Studies

Male and female *NOD.Cg-Prkdcscid Il2rgtm1Wjl/SzJ* (NSG) mice were bred in house and all care and use was approved by the University of Central Florida (UCF) Institutional Animal Care and Usage Committee (IACUC; #19 – 05, approved 04/08/2019). The UCF animal facility is accredited by the Association for Assessment and Accreditation of Laboratory Animal Care. A total of 50 six- to eight-week-old mice were used for pharmacokinetic studies. Blood and sciatic nerve samples from NSG mice were collected at 1, 2, 4, 8, and 24 hours after a single oral gavage of 75 mg/kg for pictilisib and 25 mg/kg for PF-3758309 both individually and combined. Drugs were delivered via 0.5% methylcellulose, 0.2% Tween 80, 10% DMSO vehicle. Ten mice (three per drug and one control receiving vehicle alone) were used at each time point. Collected samples were frozen and sent to Cyprotex (Watertown, MA, USA) for LC-MS/MS quantitation of drug levels. Pharmacokinetic calculations were performed using the PKSolver software. The plasma half-life (t½) was calculated as the time required to reduce by half the plasma concentration after reaching pseudo-equilibrium and is not the time required to eliminate half the administered dose. An additional LC-MS/MS analyses of a subset of the treated mice (see below) was conducted. This was done to determine whether PF-3758309 was accessible to the tumor neo-vasculature that can differ from that of the organ/tissue housing the tumor. Three mice from each treatment group were selected randomly at the end of the in vivo study to assess the levels of drug in plasma, intact control nerve, and tumor. Samples were collected 4h after the final oral gavage, frozen, and sent to Cyprotex for analysis.

### Orthotopic nerve allograft model

A total of 48 NSG mice (6 and 7 weeks old) were implanted with MS01-*Luc* as previously described(17, 19). Seven days post-implantation, mice were imaged with an In Vivo Imaging System (IVIS, Perkin-Elmer, Waltham, MA, USA) and peak radiance measurements (typically 8–15 minutes post-injection) were used to assign mice to treatment groups ensuring even distribution of initial tumor burden and sex between groups. All mice successfully established orthotopic MS01-*Luc* allografts. Mice were dosed daily by oral gavage starting on day 8. Mice (n = 12/group) received vehicle (0.5% methylcellulose, 0.2% Tween 80, 10% DMSO, pictilisib (75 mg/kg), PF-3758309 (25 mg/kg), or a combination of both treatments. Mice were imaged every 7 days and were euthanized after 14 days of treatment (4h after last treatment). Eight mice became ill and died or were euthanized due to weight loss per IACUC protocol (final sample size (n): vehicle: 11; pictilisib: 9; PF-3758309: 12; combination: 8). Tissue from n = 3 mice/group was collected, frozen, and sent to Cyprotex for analysis.

### Histology and Immunohistochemistry

Immediately after euthanization, contralateral control nerves and grafted nerves were re-moved, weighed, and measured (maximum length and width). Grafts were placed in 4% paraformaldehyde and fixed overnight at 4°C followed by standard paraffin processing. Samples were embedded and 5 μm sagittal sections were collected (from n = 4/group). Sections were stained with hematoxylin and eosin (H&E) or used for immunohistochemistry. Slides were deparaffinized and rehydrated. Antigen retrieval reaction was performed by heating sections in Antigen Unmasking Solution (pH = 6.0, Vector Labs, Newark, CA, USA) in a 100°C water bath for 20 minutes. Endogenous peroxide activity was blocked with BLOXALL solution (Vector Labs). After blocking for 1 h with 5% normal goat serum in PBS, primary antibodies were applied and slides were incubated overnight at 4°C. Primary antibodies used were rabbit anti-phospho-PAK1/2/3 (PA5-101019, S144, S141, S139; 1:120; Invitrogen, Waltham MA, USA), rabbit an-ti-T308pAKT (ab38449, 1:70; Abcam, Cambridge, United Kingdom), rabbit anti-Ki67 (ab16667, 1:200; Abcam) rabbit anti-cleaved caspase-3 (#9664, 1:500; Cell Signaling, Danvers, MA, USA), and rabbit anti-YAP (#14074, 1:200; Cell Signaling) Sections were then washed and the ImmPRESS HRP Anti-Rabbit IgG Polymer Detection Kit (Vector Labs) was used followed by ImmPACT DAB HRP substrate (Vector Labs) to develop a colorimetric (brown) signal. Sections were counterstained with hematoxylin and mounted with VectraMount Permanent Mounting Medium (Vector Labs). Sections were imaged with a Keyence BZ-X810, selecting several regions of interest (ROI) from each section; images were collected at 400x magnification. Staining was quantified as percent positive area using the IHC toolbox plugin for ImageJ (NIH, v1.53e). At least two sections per sample were used for each protein target, with a minimum of 4 ROIs imaged per section to account for tissue heterogeneity.

### Statistics

GraphPad Prism Version 7.04 was used for statistical analysis. One-way analysis of variance (ANOVA) was used to compare IVIS radiance fold change and tumor size between single and combination drug- and vehicle-treated groups. Two-way ANOVA with Bonferroni correction was used to compare treatment groups for cell cycle analysis and violet ratiometric membrane asymmetry assay. Parametric (ANOVA) and non-parametric (Kruskal-Wallis) tests were used for immunohistochemical quantification analysis after testing for normality. Statistical significance was set at p < 0.05.

## Results

### Schwannoma model cells demonstrate loss of viability with co-inhibition of PI3K and several parallel/intersecting signaling pathways

The PI3K/Akt pathway inhibitor combinations and their targets evaluated in this study are shown in Table S1. The EC50 of each agent tested as a monotherapy in five human and one mouse MD-SCs cell lines is presented in Table S2. Synergistic activity of drug combinations on MD-SC viability is summarized in Table S3 and Figure S1. The most active single agent in the MD-SC is omipalisib, with an EC50 of 2 nM in human MD-SCs. Synergy of was calculated using the Loewe Additivity model. Targeted co-inhibition of PI3K and PAK using pictilisib and PF-3758309 respectively induced the most synergistic loss of cell viability across three human MD-SC lines and one mouse MD-SC line (Table S3, Supplementary Figure S1).

**Figure 1:**
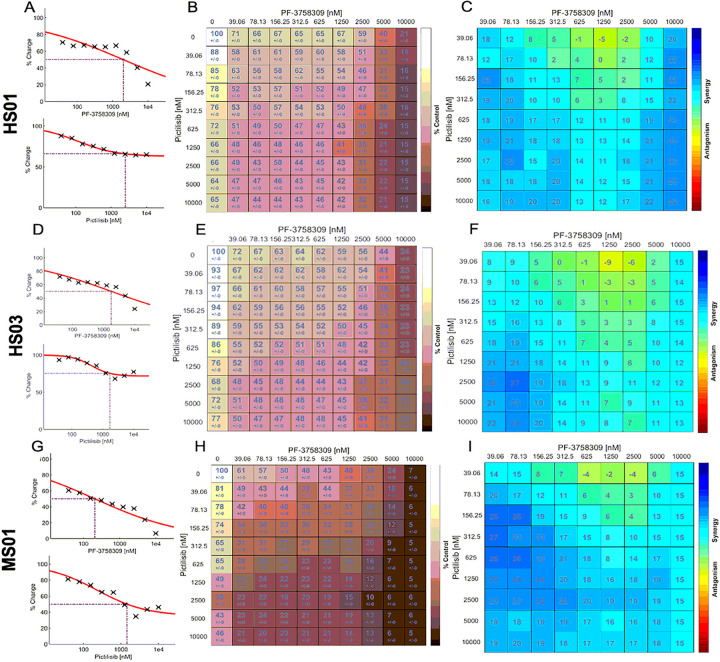
Combinatorial PI3K/PAK Inhibition Synergistically Decreases MD-SC Viability. Screening results from human MD-SCs demonstrate loss of HS01 and HS03 cells viability with single drug treatments (A and D, EC50 or ECmax indicated with dotted line), with combination drug treatment (B and E), and synergy scores for each pair of combination treatments after 72h (C and F). Similar screening performed on mouse MD-SCs shows loss of MS01 viability with single drug treatments (G), with combination drug treatment (H), and Loewe synergy scores for each pair of combination treatments after 48h (I). In B, E and H, color scale indicates loss of cell viability (light=high viability, dark=low viability) and numbers indicate percent of control cell viability. In C, F and E color scale indicates synergy (blue=synergy, red=antagonism) and numbers indicate percent difference in loss of viability compared to expected values (assuming no synergy).

Dose response results in human MD-SC: primary HS01 engineered by shRNA knockdown of merlin (isogenic normal primary HS11), and HS03 engineered by CRISPR/Cas9 deletion of *NF2* (isogenic normal primary HS13) demonstrate loss of cell viability with single drug treatments (Fig. 1A and D) and at much lower concentrations with combination drug treatment (Fig. 1B and E). Loewe synergy scores for each drug concentration combination (10×10) show consistent synergy in human MD-SCs (Fig. 1C and F). Identical screening experiments performed in mouse MD-SCs shows similar inhibitory effects of single drugs (Fig. 1G) and combination treatment (Fig. 1H). Patterns of combined treatment synergy remained consistent in mouse cells (Fig. 1I).

#### Pictilisib and PF-3558309 have different pharmacokinetic profiles alone and in combination

Blood and sciatic nerve samples were collected from mice at 1, 2, 4, 8, and 24 hours after a single oral gavage of pictilisib and PF-3758309 individually and combined. Pictilisib, when administered as a single agent, was rapidly absorbed, and distributed in both plasma and nerve (Supplemental Figure S2 A). Pictilisib penetrated the normal blood-nerve barrier (BNB), and the intraneural concentration had a longer half-life than in plasma (Supplemental Figure S2 C). Maximum plasma and nerve concentrations were reached at 1h. In contrast, PF-3758309 was not detected in the intact nerve indicating it was impermeable to the BNB, had a shorter half-life in plasma than pictilisib, and reached a maximum plasma level at 2h post oral gavage (Supplemental Figure S2 B, C). When pictilisib and PF-3758309 were co-administered, the half-life of pictilisib in plasma greatly increased compared to the monotherapy half-life (t½ 23.1h vs 4.97h, respectively, Supplemental Figure S2 D). In contrast, its half-life in nerve decreased (t½ 7h vs 16.51h, Supplemental Figure S2 F). The calculated half-life of PF-3758309 in plasma when administered together with pictilisib or alone was similar. However, the maximum concentration in plasma increased three-fold when co-administered with pictilisib (341 vs 110 ng/ml). PF-3758309 remained undetected in normal nerve when co-administered with pictilisib (Supplemental Figure S2 E). These differences support a drug-drug interaction in vivo.

#### Combinatorial PI3K/PAK inhibition decreases orthotopic MD-MSC allograft growth rate and size

To evaluate the efficacy of pictilisib and PF-3758309 in vivo, sciatic nerves of NSG mice were grafted with luciferase-expressing MD-MSCs and tumors were allowed to form for one week. Following two weeks of treatment, tumors were excised from euthanized mice. Three animals from each group were assigned for pharmacokinetic analysis, from which plasma and contralateral control (uninjected) sciatic nerves were also collected and assessed for drug concentration in each tissue at the time of euthanasia.

Concentrations of drugs were similar to those found in the initial pharmacokinetic study for plasma and nerve at 4h post-gavage (Supplemental Figure S3). However, we additionally measured the concentration of each inhibitor in the tumor, demonstrating that PF-3758309 is in fact bioavailable in the tumor despite not being detected in uninjected nerves. This suggests that the tumor neovasculature differs from that of the control nerve, and that the BNB is disrupted after nerves are grafted with merlin-deficient Schwann cells.

**Figure 2:**
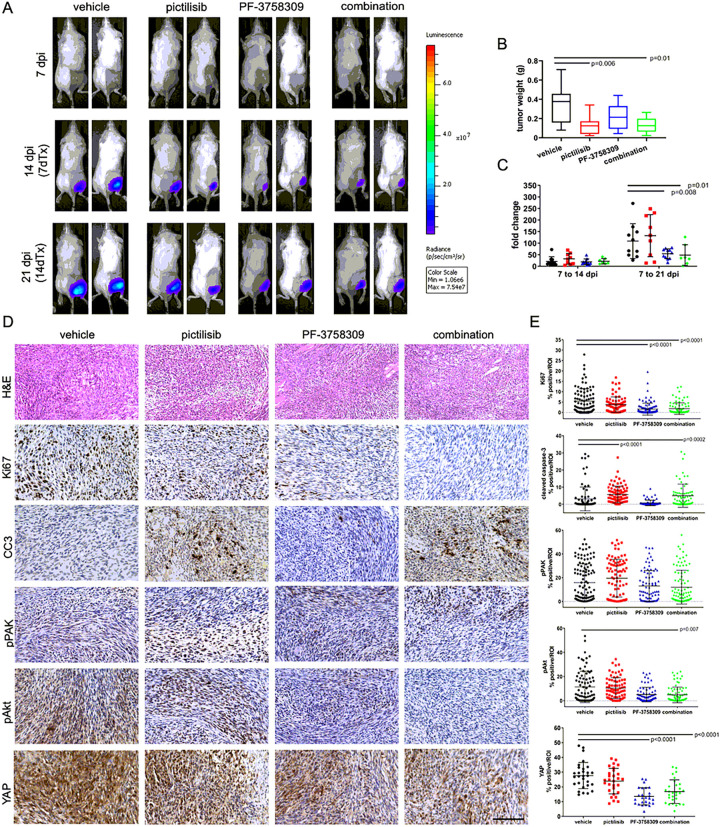
Combinatorial PI3K/PAK Inhibition Decreases Orthotopic MD-MSC Allograft Growth Rate and Weight, reduces cell proliferation, increase apoptosis, and reduces Akt phosphorylation and YAP levels *in vivo*. A. Representative bioluminescence images of signals at days 7, 14, and 21 post-cell injection (corresponds with days 0, 7, and 14 of treatment; Avg. Radiance [p/s/cm^2^/sr]). All luminescent images are shown at identical scales selected for day 21 dpi; thus, no luminescence is seen at day 7 but was present and measurable indicating successful implantation of MD-MSC. B. Tumor weight by treatment, excised tumors were significantly smaller in weight with pictilisib and combination treatments. C. IVIS imaging fold change in bioluminescent signal over treatment duration significantly decreases with both PF and combination treatments. D. Representative 400x magnification images of graft samples processed for histology and stained for hematoxylin and eosin, Ki67, Cleaved Caspase-3 (CC3), pPAK, pAkt and YAP. E. Quantification of positive IHC staining demonstrates that both PAK inhibition alone, and combination treatment reduces cell proliferation in grafts as measured by Ki67 level. Data shown as individual data points with mean ± SD. Black bars in B and C indicate post-hoc significance between treatment groups over specific study increments or at study endpoint (p<0.05). Black bars in E indicate post-hoc significance between treatment groups and vehicle controls (p<0.05). Scale bars represent 200 μm.

IVIS bioluminescent data and images demonstrate decreased rates of tumor growth with both PF-3758309 and combination treatments when assessing IVIS fold change over the treatment time course (Fig. 2A and C) All excised tumors were weighed prior to processing for pharmacokinetics or histology. Tumor weight was significantly decreased in pictilisib, and combination treated groups compared to controls (Fig. 2B).

### Combinatorial PI3K/PAK inhibition reduces cell proliferation, increases apoptosis, and reduces Akt phosphorylation and YAP expression in vivo

**Figure 3:**
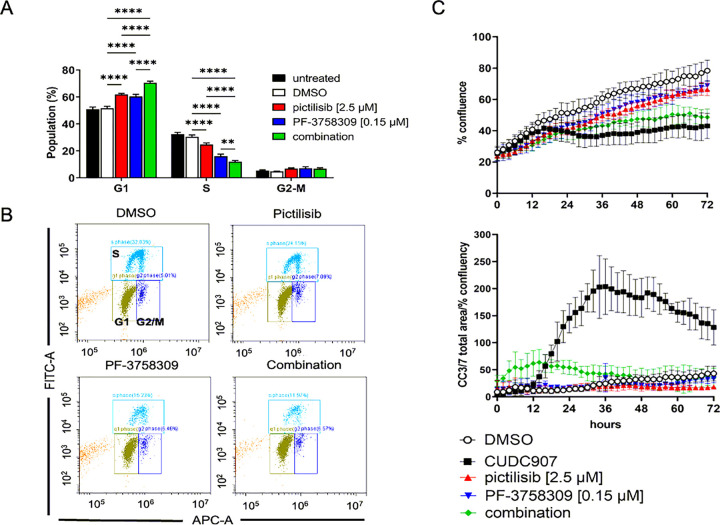
Combinatorial PI3K/PAK inhibition arrests human MD-SC at the G1 phase of the cell cycle. A. Graph of the distribution of cell cycle phases (gated for the live population) of all experimental runs as mean ± SD. **p<0.01, *** p<0.001, ****p<0.0001 B. Representative plots of the distribution of FITC (EdU) and FxCycle (DNA content)-labelled cells of 0.001% DMSO vehicle control and drug treated cells. C. Live cell imaging results of 72h treatment at EC50 (concentrations noted in legend), including cell con-fluence (top) and fluorescent cleaved caspase 3/7+ signal area normalized to percent confluency.

To evaluate specific effects of PI3K and PAK inhibition in vivo, tumors (n = 4/group) were processed for immunohistological analysis. Images of H&E-stained sections from each tumor are shown at low magnification in Supplemental Figure S4A; tumors were unique in size and shape and display regions of tissue heterogeneity. S100 was used as a marker of Schwann cells to detect grafted cells within the tumor. There were no clear differences in S100 staining patterns across groups (Supplemental Figure S4 B); this was confirmed with positive staining quantification (p = 0.86, data not shown). Ki67 staining and quantification demonstrate significantly decreased cell proliferation in PF-3758309 and combination treated groups (Fig. 2D and E). Expression of cleaved caspase 3 was increased in pictilisib and combination treated groups (Fig. 2D and E), suggesting activation of apoptotic cell death. We were unable to confirm regulation of PAK signaling, as pPAK levels were similar across control and all treatment groups (Fig. 3D and E). Additionally, inhibition of PI3K signaling as measured by pAkt levels was only significant in combination-treated samples (Fig. 2E). Because targeted pathways were not clearly inhibited with single treatments as expected, we assessed YAP as a downstream regulator of cell survival. Vehicle treated tumors stained intensely for YAP expression. Interestingly, all treatments decreased YAP staining; reduction with PF-3758309 and combination treatments were statistically significant (Fig. 2E).

#### Neither PI3K nor PAK inhibitor affect normal morphology of contralateral control nerve

Although only pictilisib can penetrate intact nerves, we assessed contralateral sciatic nerves with immunohistochemistry to qualitatively assess Schwann cell and neuron structure and morphology after exposure to the inhibitors. We were not able to distinguish differences in patterns of H&E staining or localized expression of myelin protein zero or neurofilaments (Supplemental Figure S5).

#### Combinatorial PI3K/PAK inhibition induces synergistic cell cycle arrest in human MD-SCs and mouse MD-SCs

To assess the mechanism of loss of cell viability with both pictilisib and PF-3758309, human MD-SCs were treated with single and combination drugs at EC50 concentrations. After 24h of single drug treatment, there were significantly more HS01 cells in G1 phase and significantly fewer cells in S phase (Fig. 3A and B, Supplementary Figure S6). Combinatorial treatment led to significantly larger G1 and smaller S phase populations than either single treatment. This was confirmed in a 72h time course study assessing cell confluence and cleaved caspase 3/7-positive (CC3/7+) cells. Both single treatments led to decreased cell confluence compared to DMSO controls starting at 6h of treatment (Fig. 3C, top). Combination treated cells also demonstrated significantly lower cell confluence from both control (starting at 6h) and single drug-treated cells (starting at 44h). Neither of the single drugs induced CC3/7 cleavage alone, however the combination induced low levels of CC3/7 (Fig. 3C, bottom). CUDC907 was used as a positive control for cleaved caspase 3/7-dependent apoptosis (high CC-3/7 signal starting at 14h). Representative images from several time points are shown in Supplementary Figure S7.

**Figure 4:**
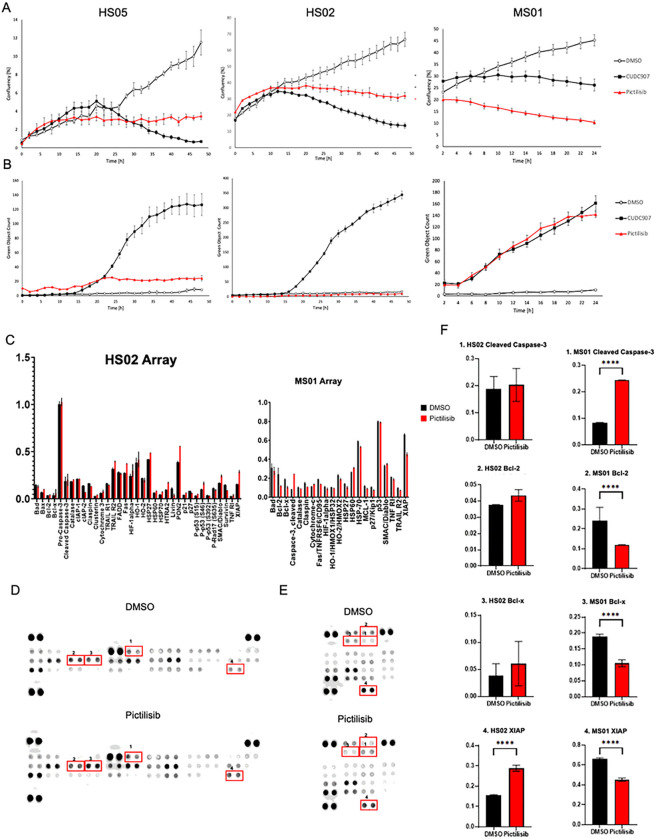
Pictilisib treatment comparison in human and mouse MD-SCs. A. Confluency measurements in two human MD-SCs (HS02 and HS05) and one mouse MD-SCs (MS01) treated with control DMSO (negative control), CUDC907 (positive control) and Pictilisib. Pictilisib significantly inhibited cell growth in all cell lines. B. Cleaved Caspase-3/7 signals for human and mouse MD-SCs treated with Pictilisib (red) and CUDC907 (black). Pictilisib significantly induced CC-3/7 signal in mouse cells but did not in human MD-SCs. C. Graphs presented all measured protein on apoptosis protein arrays, human on the right, mouse on the left. D. Human Protein Profiler Apoptotic Array representative image, numbers correspond to the proteins in the graphs. E. Mouse Protein Profiler Apoptotic Array representative image, numbers correspond to the proteins in the graphs. F. Graphs representing protein levels detected by protein arrays, numbers correspond to the spots on the arrays; *p<0.05; **p<0.01, ****p<0.0001 by Šídák’s multiple comparisons test.

Since we observed a higher cleaved caspase-3 (CC-3) signal after pictilisib treatment in the mouse model, we decided to compare human (HS02) and mouse (MS01) MD-SCs cells *in vitro* using a previously described Incucyte CC-3/7 assay(14). Human MD-SC lines exhibited significantly reduced confluency after pictilisib treatment (Fig. 4A), however no cleaved caspase-3/7 signal was detected (Fig. 4B), suggesting that pictilisib affects cell cycle but does not induce apoptosis in human MD-SCs. In contrast, mouse MD-SCs exhibited low confluency and a high CC-3/7 signal after pictilisib treatment (Fig. 4A and B). CUDC907 was used as a positive control for apoptosis. To further analyze the differences between the cell lines, we performed human and mouse apoptotic protein arrays (Fig. 4C–F). Human arrays showed no difference in CC-3, anti-apoptotic proteins like Bcl-2 and Bcl-x, and significant increase in XIAP between vehicle and pictilisib treated HS02 (Fig. 4D and F). In contrast, the mouse array showed a significant increase in CC-3 after pictilisib treatment, as well as a significant decrease in anti-apoptotic proteins Bcl-2, Bcl-x and XIAP compared to vehicle in MS01 cells (Fig. 4E and F). These results suggests that human and mouse MD-SCs respond differently to PI3K inhibition.

## Discussion

In this study we explore the synergistic effect of PI3K inhibition in combination with other kinase inhibitors and drugs on merlin-deficient Schwann cells (MD-SCs) a model for specific for Neurofibromatosis type 2-associated schwannomas. The chronic nature of NF2 and its benign characteristics make it very challenging to develop reliable and successful targeted drug treatments(20). PI3K signaling is one of the most frequently overactivated pathways in cancer. NF2 mutations lead to absent or nonfunctional merlin protein and in consequence PI3K pathway activation, making this disease a good candidate for PI3K targeted therapy(8, 21). Moreover, loss of merlin has been shown to activate other signaling pathways, including the Ras/Raf/MEK/ERK, FAK/Src, Rac/PAK/JNK, mTORC1 and Wnt/β-catenin(21). We have previously shown that simultaneous inhibition of complementary pathways exposes vulnerable targets due to synergistic effects(17). The use of high throughput viability and synergy analyses to test a large number of inhibitors was beneficial in identifying a single favorable combination that induced similar responses across a number of cell lines in multiple species. That is why we explored the synergistic effect of three PI3K inhibitors in combination with inhibitors targeting other kinases and found that the best synergy was occurred with the combination of pictilisib (pan-PI3K inhibitor) and PF-3758309 (PAK inhibitor) in human as well as in mouse MD-SCs. Pictilisib is an orally available small-molecule inhibitor for all four classes of PI3K isoforms. Human tumor xenograft models proved its anti-tumor activity with 98% and 80% growth inhibition in glioblastoma and ovarian cancer xenografts(22). First clinical phase 1 study proved pictilisib to be well tolerated with signs of anti-tumor activity in patients with advanced solid tumors(23). However, PI3K inhibitors are known to have cytostatic effects rather that cytotoxic, and their impact as single agents may primarily result in tumor stabilization rather than tumor regression. Therefore, drug combination approaches have been explored. In a phase 2 FERGI study addition of pictilisib to fulvestrant for estrogen receptor positive breast cancer patients did not significantly improve progression-free survival, and dosing of pictilisib was limited by toxicity(24). However, in another study, adding pictilisib to anastrozole significantly increased suppression of tumor cell proliferation in luminal B breast cancer(25). Combining pictilisib with the MEK inhibitor, cobimetinib in patients with advanced solid tumors had limited tolerability and efficacy despite promising pre-clinical data(26). Toxicity related to pictilisib and other pan-PI3K inhibitors might be dose dependent, thus studies that focus on the synergistic effect of PI3K inhibitors in combination with other drugs targeting complimentary pathways may result in better tolerance and effectiveness of the treatment. In our *in vivo* study, we did not observe higher mice lethality in treated groups. Animals that were lost included the vehicle group and therefore we cannot attribute their loss to drug toxicity.

P21-activated kinases (PAKs) are positioned at the nexus of several oncogenic signaling pathways. Schwannomas from NF2 patients were described as exhibiting PAK activation due to merlin loss(27). Moreover, in merlin-deficient tumors the Rac/PAK pathway was described as contributing to the loss of contact inhibition and activation of WNT/β-catenin signaling pathway(28). Later PAK inhibitors have been shown to induce schwannoma cell death via mitotic catastrophe(29). In our *in vivo* study the most significant decrease in tumor growth was observed with pictilisib and its use in combination treatment. Combination treatment presented the most significant decrease in Ki67, pAkt, and YAP levels suggesting that it had the most comprehensive influence on the tumor. In human MD-SCs most significant inhibition observed was in cell cycle with combination treatment. However, we did not observe any cleaved caspases 3 or 7 signal that was present in mouse MD-SCs after treatment with pictilisib *in vitro* as well as in the tissue. Although some data indicates that PI3K inhibitors may induce apoptosis, for example in sarcomas(30) or HER2-positive breast cancer(31), consensus is that PI3K inhibitors mostly affect cell proliferation rather than cell survival(32). That is why, to compare its influence, we examined the pictilisib-treated human and mouse MD-SCs. Here we observed that mouse and human cells respond differently to the treatment. Mouse MD-SCs undergo apoptosis with the induction of caspase 3 cleavage and Bcl-2 and Bcl-x downregulation. Human MD-SCs cells did not present with cleaved caspase 3 or downregulation of anti-apoptotic proteins with pictilisib treatment. Indicating that assumptions and pre-clinical data obtained using mainly animal models should be validated using human cells, especially in the area of NF2 schwannoma research where designing animal models of slowly growing schwannomas is a challenge.

Comprehensive pharmacokinetic assessments determined the time course of the presence of drugs in blood plasma and nerve tissue suggesting drugs should be present at 4h post-gavage. However, metabolism can vary due to a number of environmental factors(33), and detection of a drug does not always correlate with its activity. However, we demonstrated that co-treatment alters drug metabolism and increases the half-life of pictilisib in plasma, which may be a mechanism of drug synergy and explain the increased PI3K inhibition in combination-treated animals. Further studies could elucidate drug activity in relation to the presence of these inhibitors during metabolism.

We identified clear changes in YAP expression in PF-3758309 and combination treated tumor tissue. The Hippo pathway plays an essential role in maintaining tissue homeostasis by regulating cell processes such as proliferation, migration, and survival. Merlin is a key upstream regulator of the Hippo kinase core protein, suppressing downstream YAP activity as a transcription factor(34). Not surprisingly, loss of merlin leads to dysregulation of YAP activity with its co-transcription factors TAZ and TEAD(34). The ability to block YAP/TAZ function has been of interest in treating NF2-deficient cancers(35). A number of studies have targeted YAP association with TEAD as a specific method to suppress its activation and role in tumor formation(36). Our study presents a novel method through which overall YAP expression is reduced; further studies should clarify the mechanism of these pathway interactions.

In summary, we demonstrated that targeting PAK and PI3K pathways provide synergistic activity in several models of NF2, leading to cell cycle arrest or initiation of apoptosis. Our study uncovered another anti-tumor mechanism for schwannoma treatment. Understanding these mechanisms can improve identification of new targets and inhibitors to slow NF2 schwannoma disease progression. Furthermore, these results support the use of combinatorial treatments to take advantage of cooperative properties of targeted inhibitors.

## Data Availability

Data sharing not applicable to this article as no datasets were generated or analysed during the current study.

## Abbreviations

AKT: protein kinase B
ALK: anaplastic lymphoma kinase
ANOVA: analysis of variance
BNB: blood-nerve barrier
CC3/7: cleaved caspase 3/7
DMSO: dimethyl sulfoxide
EGFR: epidermal growth factor receptor
FAK: focal adhesion kinase
H&E: hematoxylin and eosin
HRP: horseradish peroxidase
IHC: immunohistochemistry
IVIS: in vivo imaging system
LC-MS: liquid chromatography-mass spectrometry
Luc: luciferase
MAPK: mitogen-activated protein kinase
MD-SC: merlin deficient Schwann cell
mTOR: mammalian target of rapamycin
NF2: neurofibromatosis type 2
NSG: NOD.Cg-Prkdcscid Il2rgtm1Wjl/SzJ
PAK: p21 activated kinase
PI3K: phosphoinositide-3 kinase
ROI: region of interest
SC: Schwann cell
SCM: complete Schwann cell media
VEGF: vascular endothelial growth factor
VS: vestibular schwannoma
YAP: yes-associated protein
